# The Role of Health Literacy in Gastrointestinal Cancer Patients’ Surgical Journey: A Qualitative Study

**DOI:** 10.1097/AS9.0000000000000643

**Published:** 2026-01-14

**Authors:** Bayley A. Jones, Gianina C. Hernandez-Marquez, Luke Mondia, Ivan I. Herbey, Burkely P. Smith, Nataliya V. Ivankova, Wendelyn M. Oslock, Nathan English, Yu-Mei Schoenberger, Maria Pisu, Daniel I. Chu

**Affiliations:** From the * Department of Surgery, University of Alabama at Birmingham, Birmingham, AL; † School of Health Professions, University of Alabama at Birmingham, Birmingham, AL; ‡ Department of Quality, Birmingham Veterans Affairs Medical Center, Birmingham, AL; § Department of General Surgery, University of Cape Town; ‖ Division of General Internal Medicine and Population Science, O’Neal Comprehensive Cancer Center, University of Alabama at Birmingham, Birmingham, AL

**Keywords:** gastrointestinal cancer, health literacy, qualitative, surgical cancer care

## Abstract

**Objective:**

Examine the role of low health literacy in surgical cancer care.

**Background:**

Disparities exist in surgical cancer care in the Deep South, focused here on the states of Alabama and Mississippi. Low health literacy is prevalent in this region and is associated with worse surgical outcomes.

**Methods:**

We conducted semi-structured interviews with gastrointestinal cancer patients and providers to explore the influence of health literacy on the surgical journey. Participants were recruited using a purposeful sampling with a snowball-recruitment approach. Verbatim interview transcripts were coded with NVivo 12.6 Plus using inductive thematic analysis to develop a codebook, followed by content analysis of the coded data. A constant comparative method was employed to ensure that saturation in the data was achieved. The inter-coder agreement was established at the recommended 90%.

**Results:**

Thirty-six patients and 32 providers were interviewed, including 15 surgeons. In the preoperative phase, low health literacy contributed to patients’ difficulty in understanding diagnosis, facilitating their own care, understanding costs/insurance, and delays in care. In the perioperative phase, low health literacy increased difficulty understanding treatment plans and created barriers to compliance with staged treatments. In the postoperative phase, low health literacy led to difficulty understanding complications and the need for more follow-up. Common subthemes included difficulty following instructions and the usefulness of visual aids and teach-back methods.

**Conclusions:**

Cancer providers and patients highlighted the role of health literacy in all phases of the surgical journey, particularly in creating barriers to understanding key components of cancer care. Interventions to address these barriers will be critical to improve care for low health literacy patients.

## INTRODUCTION

Gastrointestinal (GI) cancers represent a quarter of all cancer cases worldwide and one-third of cancer-related deaths.^1^ Disparities in GI surgical cancer care exist with vulnerable patient populations, such as Black and rural patients, being less likely to receive standard-of-care treatments amid higher cancer incidence, late-stage presentations, and cancer-related mortality.^2–5^ Although these cancer disparities may be explained in part by biological factors, studies have demonstrated the important role that social determinants of health, including health literacy, play in driving outcomes and disparities.^6–8^

Health literacy is defined as the ability of an individual to obtain, understand, and apply information to maintain and improve their health.^9^ Over one-third of surgical patients may be impacted by limited or low health literacy, and Black patients experience limited health literacy at almost 3 times the rate of white patients.^10,11^ Patients with low health literacy face challenges in accessing, understanding, and using information to make decisions about their health. This has been shown to result in longer length-of-stays, more frequent complications, and higher rates of readmissions compared to patients with adequate health literacy.^12–14^ Prior qualitative studies have examined providers’ perspectives of health literacy barriers, revealing concerns regarding time constraints in communicating with patients during busy clinical settings, lack of educational materials, and patient shame (eg, fear of exposing their literacy level).^15^ However, no studies have examined providers’ and patients’ perspectives of how health literacy may impact surgical cancer care, particularly in the Deep South—a region that experiences some of the highest rates of low health literacy in the country.^16^ The Deep South, a region known for including the states of Mississippi, Alabama, Georgia, Louisiana, and South Carolina, with Mississippi and Alabama being at its core. These have been commonly associated with poor health outcomes, socioeconomic status, and historically relied on their economy on cotton agriculture.

To address this knowledge gap, we aimed to assess the perspectives of providers, cancer care team members, and patients on how health literacy may impact the surgical journey for patients undergoing GI cancer care. Using qualitative interviews, we aimed to identify perceived barriers created by low health literacy throughout the surgical journey, including preoperative, perioperative, and postoperative phases of care.

## METHODS

This study is part of a larger mixed methods study, Advancing Surgical Cancer Care and Equity in the Deep South, aimed at identifying gaps in surgical care for GI cancers (specifically, esophageal, pancreatic, and colorectal cancer) in the Deep South. The study was approved by the University of Alabama at Birmingham Institutional Review Board (IRB-300005475). Health care personnel and patients gave consent for the interview and received a monetary incentive.

### Participants and Recruitment

Health care personnel included adults 18 years old or older who provided care for GI cancer patients. A list of GI cancer surgeons in Alabama and Mississippi was compiled, and they were contacted via email. Using a snowball recruitment approach, we asked these participants to identify and recommend other team members or colleagues for us to approach for an interview.^17^ We also recruited participants by asking patients who participated in the study if they would identify their providers or the clinics where they received care. Although the focus of this study was on surgical cancer care, other providers, such as oncologists, were included to provide a more encompassing multidisciplinary perspective.

Patients included adults (aged ≥18 years) diagnosed with stage I to III esophageal cancer, pancreatic cancer, or colorectal cancer within the past 3 years; were English speaking; and were able to participate in a 1-hour interview. Hospital registries and the Alabama Statewide Cancer Registry served as the sources for recruitment. A letter describing the study’s purpose and contact information was mailed to potential participants. Five or more days thereafter, these patients were contacted via phone by recruiters from the O′Neal Comprehensive Cancer Center Participant Recruitment and Assessment shared facility. For broader expansion of recruitment, the Advancing Surgical Cancer Care and Equity in the Deep South team also partnered with other institutions in Alabama and Mississippi, who proceeded to contact patients via mail, phone, or in person before releasing contact information to the study team for potential recruitment.

### Interview Guide and Process

The interview guides for health care personnel and patients were developed based on 2 frameworks: a surgical disparities framework and an access to care framework. The surgical disparities framework conceptualizes patient, provider, system, and other factors that lead to surgical disparities along the surgery continuum.^18,19^ The access to care framework characterizes access by 5 domains, including availability, accessibility, accommodation, acceptability, and affordability.^20–22^ Quality surgery, defined by these 5 domains, depends on patient, provider, and system factors highlighted by the surgical disparities framework.^23^

The structure of the health care personnel’s interview guide focused on the preoperative, perioperative, and postoperative phase of surgical care (Supplemental Table 1, https://links.lww.com/AOSO/A563). Questions addressed factors that were barriers or facilitators to access to surgery. Participants were asked to describe typical patient experiences and share examples of atypical patient experiences. Probing questions differed slightly between surgeons and other personnel.

The structure of the patient’s interview guide focused on the preoperative, perioperative, and postoperative phase of surgical care (Supplemental Table 2, https://links.lww.com/AOSO/A563). These interviews were conducted over the phone by a trained interviewer from the O′Neal Comprehensive Cancer Center Participant Recruitment and Assessment facility who had extensive experience conducting interviews with cancer patients.

In addition to participating in interviews, each patient’s level of health literacy was assessed using the Brief Health Literacy Screening Tool Health Literacy Screening Tool. This screening tool is a validated 4-item questionnaire designed to rapidly assess a patient’s health literacy in clinical settings. The 4 questions are rated on a 5-point Likert scale, and the total score ranges from 4 to 20 (4–12 categorized as inadequate health literacy, 13 to 16 as marginal health literacy, and 17 to 20 as adequate health literacy).^24,25^ These were conducted before the participants were interviewed, and they were self-reported on the survey.

### Data Analysis

All interviews were recorded and transcribed by an independent commercial transcription company. Transcripts were verified using the audio files for transcription accuracy and analyzed with NVivo 12.6 Plus (Lumivero)^26,27^ first using an inductive thematic approach to identify common themes and related subthemes in the data^26^ followed by content analysis on the generated themes and codes to systematically represent consistencies and variations in participants’ viewpoints. A constant comparative method that involves iterative comparison of new information with already coded data^28^ was employed to ensure saturation,^29^ meaning that no new participants’ perspectives across the sample were missed. An inductive thematic analysis helped develop themes that describe providers’ and patients’ perspectives of the role of perceived health literacy in each phase of the surgical journey, while subsequent content analysis helped better understand how limited health literacy may have contributed to perceived barriers that shaped participants’ surgical journey experiences.

Three researchers (I.I.H., B.P.S., and N.V.I.) with extended experience in qualitative data analysis independently analyzed the transcripts in batches of 2 transcripts and met to discuss the emergent codes and themes to resolve discrepancies in coding and to develop the codebook. They also regularly met with the rest of the research team to discuss the emergent themes and to further refine the codebook. The inter-coder agreement was calculated at each meeting, targeting the recommended 90% consensus to ensure quality of data interpretation^30^

## RESULTS

### Participant Characteristics

Participant characteristics are detailed in Table 1. For health care personnel, 32 interviews were completed. We attempted to contact 81 providers. Of those, 1.2% had disconnected phones, 60.5% not reached, 4.9% other (ie, opted out or did not show for the interview), and 33.3% completed the interview. Participants who completed interviews included providers: surgeons (n = 15), medical oncologists (n = 2), radiation oncologists (n = 2), a primary care physician (n = 1), and team members: nurses (n = 9) and patient navigators (n = 3). The mean age of participants was 42.8 years, and the majority were white (n = 19, 59.4%). The most represented cancer types were pancreatic (n = 7, 21.9%) and colorectal (n = 7, 21.9%), followed by esophageal (n = 6, 18.8%), although many participants provided care for more than 1 cancer type (n = 11, 34.4%). The majority of personnel were from Alabama (n = 25, 78.1%), and 7 participants (21.9%) were from Mississippi. Most were from urban areas (n = 19, 70.4%).

**TABLE 1.** Participants Demographics

	Providers N = 32	Patients (n = 36)
Age, mean (range)	42.8 (24.6–74.1)	64.16 (47.0–87.0)
Race, N (%)		
White	19 (59.4)	24 (66.7)
Black	3 (9.4)	11 (30.6)
Other	8 (25)	1 (2.8)
Missing	2 (6.3)	0 (0.0)
Gender		
Female	14 (43.8)	15 (41.7)
Male	18 (56.3)	21 (58.3)
Provider type, N (%)		
Surgeon	15 (46.9)	N/A
Nurse	9 (28.1)	N/A
Patient navigator	3 (9.4)	N/A
Medical oncologist	2 (6.3)	N/A
Radiation oncologist	2 (6.3)	N/A
Primary care physician	1 (3.1)	N/A
Cancer type, N (%)*		
Pancreatic	7 (21.9)	7 (19.4)
Esophageal	6 (18.8)	10 (27.8)
Colorectal	7 (21.9)	19 (52.8)
Combination	11 (34.4)	N/A
State, N (%)		
Alabama	25 (78.1)	19 (52.8)
Mississippi	7 (21.9)	17 (47.2)
Rurality		
Urban	22 (68.8)	11 (30.6)
Rural	8 (25)	23 (63.9)
Other	2 (6.3)	2 (5.6)
Health literacy level, N (%)†		
Inadequate (4–12)	N/A	9 (33.3)
Marginal (13–16)	N/A	15 (55.6)
Adequate (17–20)	N/A	3 (11.1)

* For cancer type, 6 providers indicated they focus on all 3 cancers, and 4 providers indicated they focus on colorectal and pancreas.

† For Health Literacy Level, 9 patients did not complete the Brief Health Literacy Screening Tool health literacy assessment; hence, the sample used for calculations was n = 27.

Thirty-six GI cancer patient interviews were completed. We attempted to contact 178 survivors. Of those, 5.1% had disconnected phones, 39.3% not reached, and 22.5% other (eg, deceased, incapable, or ineligible). Of the remaining 33.1%, 20.2% completed the interview, and 12.9% refused. The mean age of participants was 64.2 years, and 58.3% were male. Twenty-four patients were identified as white (n = 24, 66.7%) and 11 as Black (n = 11, 30.6%). Colorectal cancer (n = 19, 52.8%) was the most represented cancer type, followed by esophageal (n = 10, 27.8%), then pancreatic (n = 7, 19.4%). Most of the patients were from Alabama (n = 19, 52.8%), and 17 (47.2%) were from Mississippi. Regarding health literacy level acquired from the Brief Health Literacy Screening Tool survey, 15 patients fell under the marginal category, 9 under inadequate, and only 3 had an adequate level of health literacy.

### The Role of Health Literacy in the Surgical Journey for Gastrointestinal Cancer Patients

**FIGURE 1.**
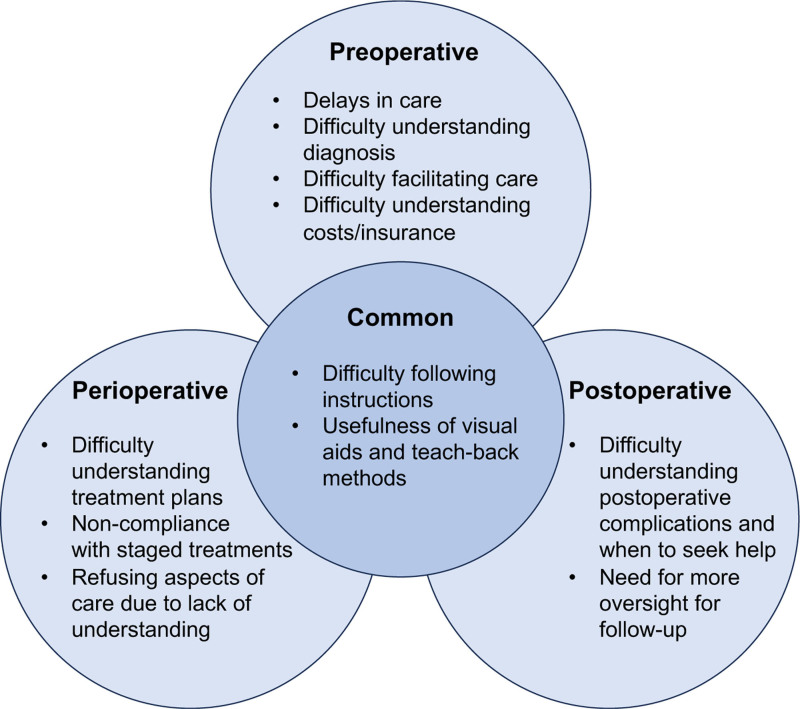
Subthemes for each phase of the surgical journey.

Three main themes emerged reflecting the role of health literacy in the 3 phases of the surgical journey: preoperative, perioperative, and postoperative (Fig. 1). Figure 1 illustrates the subthemes that emerged in each part of the surgical journey as well as the common subthemes across the 3 phases. Themes, subthemes, and additional representative quotes are outlined in Table 2. The following major themes and subthemes were identified: (1) Theme 1: Low health literacy creates barriers in the preoperative phase, with subthemes delays in care, difficulty understanding diagnosis, difficulty facilitating care, difficulty understanding insurance/costs, difficulty implementing instructions, and usefulness of visual aids and teach-back methods, (2) Low health literacy creates barriers to compliance in the perioperative phase, with the subthemes difficulty understanding treatment plans, refusing aspects of care, difficulty implementing instructions, and usefulness of visual aids and teach-back methods, and (3) Low health literacy impacts postoperative care with subthemes difficulty understanding postoperative complications, difficulty implementing instructions, usefulness of visual aids and teach-back methods, and need for more guidance during follow-up.

**TABLE 2.** Qualitative Themes, Subthemes, and Illustrative Quotes Regarding Health Literacy and Gastrointestinal Cancer Surgical Care

Subthemes	Illustrative Quote(s) Providers	Illustrative Quote(s) Patients
Theme 1. Low health literacy creates barriers in the preoperative phase		
Delays in care	“If you don’t understand that having difficulty swallowing is unusual, and you should go see the doctor right away, and you wait 6 months, well, you present in a later stage of disease, right.” (Surgeon 22, male, esophageal)	“…I didn’t know I was sick. I didn’t know I had cancer. It had been going on for weeks. I didn’t know. My stomach was hurting me so bad. But it would go and stop and go and stop…” (Patient 11, female, rural, marginal health literacy)
Difficulty understanding diagnosis	“…if you have someone that has never been exposed to anything medical or may not have a true understanding of like the basics … it can be a little bit difficult for them to understand.” (Nonsurgeon 12, female, pancreatic)	“…I don’t know anything about cancer, so I started researching my path, trying to understand my pathology report. Of course, adenocarcinoma was all I could see and so in my head, I had already limited my life to 5 years, so you just kind of Google everything and you’re trying to gather that information and make sense of it.” (Patient 33, female, rural, marginal health literacy)
Difficulty facilitating care	“Some of it is just education. Some of it is understanding how to prioritize what matters…some people don’t get it filled out and then are hard to track down.” (Surgeon 23, male, esophageal)	
Difficulty understanding insurance/costs	“…don’t know common…deductible, copay, max out-of-pocket cost, the differences in those things. Across the board, I find that peoples’ insurance health literacy is very low.” (Nonsurgeon 20, female, colorectal)	“I told you I have a learning disability anyway and it’s just very confusing to me…they’ll send 4 bills for the same doctor and all 4 bills will come at the same day with the same doctor’s name on it in separate envelopes. How does that make sense when they get to send one bill for that doctor?” (Patient 9, female, rural, unknown health literacy)
Difficulty implementing instructions	“It’s a huge problem. Health literacy is a huge problem… 30 minutes of talking and I feel like that they didn’t understand a single word I’ve said and I don’t even know how to get them to understand…” (Surgeon 7, male colorectal)	“…it was very simple; it was very easy to understand; they were just simple. “Do this; do this; do this; do this; do this,” and the exact order it should be done in and that’s what I did, and it went well.” (Patient 9, female, rural, unknown health literacy)
Usefulness of visual aids and teach-back methods	“I go through it with the patients and I draw and write in things kind of as needed for those who are of a lower literacy level…The pictures probably help more in those circumstances than the written part…” (Surgeon 23, male, esophageal)	“…he went as far as to draw me a diagram with a pen and piece of paper to let me know exactly what he was going to do, exactly why he needed to do it; and what he thought the outcome would be from him doing it. I still have the piece of paper with the diagram on it. So, I mean he drew out exactly what he’s got to cut out, exactly what it’s going to look like, and everything.” (Patient 9, female, rural, unknown health literacy)
Theme 2. Low health literacy creates barriers to compliance in the perioperative phase		
Difficulty understanding treatment plans	“Just understanding the complexity of…going through a staging algorithm on getting one procedure and then the next procedure and being compliant with all that…” (Surgeon 22, male, esophageal)	“He didn’t really--I don’t know if he gave me any other option--only option he gave me he said it needed to be taken out.” (Patient 4, female, urban, unknown health literacy)
Refusing aspects of care	“One of the strongest things that we emphasize…the perioperative anesthetic block…many patients refuse saying that they just don’t want to go through another procedure or they’re afraid of needles…” (Surgeon 4, male, pancreatic)	“Like I said, the main thing for me is it goes back to the so-called plan of care, which is nonexistent as far as the patient is concerned. Because you’re not involved in it really, and you don’t know what the final thing is; the plan of care is not given to the patient so that you know, basically, who is on the team, what they’re going to do, at what point do you have scans taken? Because that was another thing that ticked me off, being given a scan when I didn’t want a scan; the oncologist ordered it but I didn’t want one. And then, because the surgeon’s is going to order scans and the oncologist is going to order scans, the patient should have a right to say…” (Patient 4, female, urban, unknown health literacy)
Difficulty implementing instructions	“…those kinds of barriers where it’s like the patient seems to understand, but when it comes to like hospital stay or some of the self-care things after the surgery… they don’t understand or they may even say, ‘Well, that wasn’t explained to me.’” (Nonsurgeon 12, female, pancreatic)	
Usefulness of visual aids and teach-back methods	“I require the patient to respond to--recall back to me all the complications that could potentially happen during surgery…and if they’re having trouble with that, I make sure they write that in their notebook as well. And then it’s time to operate.” (Surgeon 4, male, pancreatic)	“They explained everything to me slowly. They kept going over and over again. They made sure I understood the whole thing.” (Patient 5, male, rural, unknown health literacy)
Theme 3. Low health literacy impacts postoperative care		
Difficulty understanding postoperative complications	“…they don’t understand the seriousness of, ‘Hey, I have fevers and chills and my incision is oozing; I need to get to the hospital quickly.’” (Nonsurgeon 12, female, pancreatic)	“I wish I could say a lot of how to make it better because it was a rough surgery and they told me it was going to be a rough surgery before they done it. But not being able to eat? See, I lost three months without being able to eat anything.” (Patient 5, male, rural, unknown health literacy)
Difficulty implementing instructions	“Unfortunately, you still have some of those lower-education people who they say that they understand and that they get it, but unfortunately, after surgery, it’s proven otherwise, they can’t follow the simple instructions correctly.” [*regarding surgical drains*] (Nonsurgeon 12, female, pancreatic)	
Usefulness of visual aids and teach-back methods	“I find that they understand the complications a little bit better since I’ve made them recall it back to me; I find that they hold on to better retention in that situation. I feel that patients get overwhelmed when it comes to understanding their immediate postoperative instructions.” (Surgeon 4, male, pancreatic)	“Very easy. It at all. Because everything was laid out in paper. Just follow the instructions went over with me like all 10 of them…I followed the instructions very easy for me to understand because they kept going over and over it. Repeating themselves to make sure I did understand.” (Patient 2, male, urban, unknown health literacy)
Need for more guidance during follow-up	“I think for patients who are a little more isolated, rural areas, a little more limited in their capacity, I think they need a shorter follow-up. I often see them back in a week…” (Surgeon 7, male colorectal)	

### Theme 1: Low Health Literacy Creates Barriers in the Preoperative Phase

Before any surgical intervention, providers and patients perceived low health literacy as contributing to delays in seeking care at the onset of symptoms. Providers felt that low health literacy patients did not recognize abnormal signs and symptoms to know when they should seek medical attention. Patients supported this, explaining that they had difficulty understanding the potential implications of their symptoms, which led to delays in care.

“…over a six-month period, I kind of knew something was going on my body before I went to the doctor, I just wasn’t sure what…I have a dual diagnosis of disability; I have problems sometimes understanding or remembering what’s been done to me or just grasping the reality of what the situation.” (Patient 9, female, rural, unknown health literacy).

Providers and patients also perceived difficulties in understanding diagnoses. Providers felt that low health literacy made it difficult for patients to understand and comprehend their cancer diagnoses.

“…spend 20 minutes talking about the diagnosis of cancer…Many are already limited in their health literacy and their own educational background. They’re already limited so how much can they grasp...” (Surgeon 7, male, colorectal).

Patients felt it was difficult for them to understand the severity of the diagnosis, and that the lack of understanding led to jumping to conclusions and relying on internet resources to try to understand what they were facing.

“…I don’t know anything about cancer, so I started researching my path, trying to understand my pathology report. Of course, adenocarcinoma was all I could see and so in my head, I had already limited my life to five years, so you just kind of Google everything and you’re trying to gather that information and make sense of it.” (Patient 33, female, rural).

Providers felt that low health literacy created a barrier for patients to play an active role in facilitating their care. This included tasks such as obtaining and sending health records to their physicians or completing necessary paperwork.

“…if you have a patient that is health literate, and very proactive in general…they could potentially be on the ball saying, ‘Send my records to this, this, and this.’ But most of the patients…just simply, the health literacy is not there.” (Nonsurgeon 6, female, pancreatic cancer).

Providers and patients felt that low health literacy made it difficult for patients to understand insurance policies and costs related to their care. Providers felt that these patients do not understand the differences in deductibles and out-of-pocket costs. Patients expressed confusion related to bills sent from physicians and hospitals, as well as coverage included in their insurance plans.

### Theme 2: Low Health Literacy Creates Barriers to Compliance in the Perioperative Phase

In the perioperative phase, providers felt that patients with low health literacy had difficulty understanding the treatment plan for their cancer care. This resulted in patients not understanding the need for (or lack thereof) different procedures and steps of treatment.

“…I think the less healthcare-literate folks will say, ‘No, I have cancer; cut it out.’ We have this thing in Mississippi where the really, really healthcare-illiterate folks will say, ‘No, you can’t operate on me because if you expose the cancer to air, it’ll make it spread.’” (Surgeon 27, male, pancreatic).

This lack of understanding was mirrored in patients not remembering the nuances of discussions about treatment options.

Providers also felt that patients with low health literacy sometimes refuse aspects of care, such as regional pain blocks, likely due at least in part to the lack of understanding of the benefits. Patients expressed frustration at not understanding aspects of their treatment plans, such as the importance of scheduled pain medications, such as Tylenol, which led to refusal of the medication.

“I’m not a person to take pain medication, because I want to know I got pain. Because, to me, if I got pain, there’s something wrong…I decided not to take Tylenol…I don’t want the pain masked and I don’t know that it’s building up.” (Patient 4, female, urban, unknown health literacy).

### Theme 3: Low Health Literacy Impacts Postoperative Care

In the postoperative phase, providers felt that patients with low health literacy had difficulty recognizing postoperative complications and knowing when to notify the surgical team. Providers explained that patients may not recognize the severity of symptoms and often have difficulty explaining their symptoms when speaking to care team members.

“…it’s the health literacy because sometimes you’re trying to describe to a patient or have them describe to you what’s going on, and they can’t seem to put it into words.” (Nonsurgeon 5, female, pancreatic).

In addition, patients often may not understand the importance of making their surgical care team aware of issues. Patients did not perceive that postoperative expectations were clearly established, and did not understand that certain symptoms could be a complication of surgery.

Providers and team members felt that, oftentimes, patients with low health literacy need more oversight regarding follow-up postoperatively. As a result, the office will schedule follow-up sooner so that patients do not get lost to follow-up.

“When they leave the hospital, our office is notified to set up the follow-up appointment. So, we try to go ahead and make that--we ask them to call, but we try not to leave it dependent on them calling because some people, we may not ever see again.” (Nonsurgeon 5, female, pancreatic).

### Common Subthemes Across All Operative Phases

Providers felt that patients with low health literacy have difficulty following instructions in the preoperative, perioperative, and postoperative phases. Preoperatively, providers felt that patients have difficulty following instructions needed to optimize comorbidities or nutrition. However, patients did not express difficulty with preoperative instructions and felt that they were simple and easy to understand. Perioperatively, providers reported concerns with inadequate bowel preparation and self-care hygiene practices.

“…preparation of the colon for surgery is very important…and they may or may not take the pills…take all of the laxative; they may or may not shower… (Surgeon 26, male, colorectal).

Postoperatively, providers reported that difficulty following instructions, such as caring for surgical drains, is a significant problem.

### Potential Strategies to Deal and Address Patient’s Low Health Literacy

Providers and patients both felt that visual aids and teach-back methods were important educational tools to help patients with low health literacy during their surgical journey. Providers felt that repetition, teach-back, and using pictures were helpful in their encounters with patients in all surgical phases. Patients particularly appreciated drawings and repetition to help them understand various aspects of their diagnoses, treatment plans, and care.

“I think at the visit itself, we need some way to educate people who have lower health literacy. We need some support to make sure patients with lower health literacy really understand what it is we’re doing. Either through education guides, teaching modules, something. We need some support. We need something there to help us to offload some of the work and some of the burden from us and the nurses, from the physicians and the nurses.” (Surgeon 7, male, colorectal).

“I’ll never forget it. And he drawed me the picture, showed me what it was and what the deal was and explained everything to me.” (Patient 13, female, N/A, inadequate health literacy).

## DISCUSSION

In this qualitative study of GI cancer care providers and patients in the Deep South, we identified the impact of low health literacy on all phases of the surgical journey. In the preoperative phase, patients with low health literacy have delays in care, difficulty understanding their diagnosis, difficulty facilitating their own care, and difficulty understanding insurance and costs. In the perioperative phase, they have difficulty understanding treatment plans, which sometimes leads to refusal of care. Finally, in the postoperative phase, these patients have difficulties in recognizing postoperative complications, and they often need more guidance during follow-up care. Across all 3 phases of the surgical journey, providers highlighted that patients face difficulties understanding instructions, and both providers and patients acknowledged the usefulness of visual aids and recall in efforts to overcome these barriers. Overall, our findings provide a better understanding of the ways that health literacy impacts GI surgical cancer care, which can inform patient-centered interventions to improve care for these patients.

Low health literacy was identified by providers as an important factor contributing to barriers to understanding key aspects of surgical cancer care. This is consistent with prior studies that have shown an association between health literacy and patient understanding of verbal communication and written instructions.^31,32^ Chu et al^33^ found that patients with higher health literacy had improved understanding of preoperative information, a relationship strengthened among patients who perceived greater empathy from their physicians. Studies have also demonstrated that health professionals often have a limited understanding of health literacy, which impacts patient care.^34,35^ Gibson et al^36^, showed that an interactive health literacy training intervention for primary care staff improved staff’s knowledge and behavior in using health literacy strategies with patients. Providing surgical cancer care providers with health literacy training may improve communication and education to help patients with low health literacy better understand important information related to their care. Based on our findings, we suggest that incorporating visual aids, repetition of instructions, and teach-back methods into such training programs may be an effective way to better care for these populations.

Limited patient understanding also contributes to difficulty with treatment plan compliance, difficulty facilitating care, and the need for more touchpoints during follow-up. Notably, low health literacy has also been shown to be associated with patients not agreeing to surgical treatment. For example, patients with low health literacy were less likely to agree to bariatric surgery and surgery for urinary incontinence when indicated.^37,38^ Patzner et al^39^, showed the important association of low health literacy and medication nonadherence in patients who have undergone kidney transplantation, which is a critical part of postoperative care.^39^ Similarly, in our study, providers suggested that patients with low health literacy were sometimes noncompliant with cancer staging protocols and had fundamental misunderstandings about the risks of undergoing surgery. Patients, on the other hand, reported that providers and team members failed to educate them on treatment options. Patients also highlighted not feeling part of the “plan of care.” Both scenarios may contribute to noncompliance with treatment plans.

Two common subthemes emerged in all 3 surgical phases: difficulty following instructions and the use of recall and visual aids to improve understanding. Studies have repeatedly demonstrated the association between health literacy and the ability to implement instructions. Chew et al^40^, found that low health literacy was associated with lower adherence to preoperative medication instructions.^40^ A study of cardiac surgery patients showed a positive correlation between health literacy and understanding discharge instructions, in addition to instructions being well above the reading level of the average patient.^41^ In our study, many providers suggested that repetition, patient recall, and visual aids, such as drawings, were effective in helping to increase understanding and thus patients’ ability to follow instructions. Patients in our study also highlighted the importance of visual aids and repetition of instructions, which enabled them to better understand what their treatment would entail. The importance of patient recall or using the teach-back method has been demonstrated in patient communication, especially in patients with low health literacy.^42,43^ Furthermore, the use of visual aids, such as pictures and drawings, has also been shown to improve understanding of instructions among patients with low health literacy.^44,45^ Provider education utilizing these methods during patient communication may help patients with low health literacy follow instructions before, during, and after surgery. Further research is needed to develop health literacy-sensitive implementation strategies to improve patient education and engagement.

In addition to comprehension and application barriers, providers in our study suggested that patients with low health literacy have difficulty facilitating their own care. Providers specifically highlighted difficulty completing paperwork and billing components, steps that, if missed, can delay treatment. Patients felt similarly as they reported lacking understanding of how to deal with insurance and costs. Lane et al^46^, demonstrated that the majority of patients with low health literacy reported difficulty filling out necessary paperwork. Finally, providers in our study also highlighted concerns regarding low health literacy patients’ inability to not only detect complications but also describe the potential complications they are experiencing, thus requiring more robust follow-up strategies. Similarly, Hecht et al^47^ found that low health literacy was the only variable related to missed bariatric appointments, highlighting the additional supports that may be needed to overcome this barrier.

There are several limitations to our study. First, this study only included GI cancer providers and GI cancer patients, so findings may not be applicable to all cancer types. Second, participating clinicians and patients were from 2 states in the Deep South: Alabama and Mississippi. This area has some of the highest rates of low health literacy in a racially diverse and rural population; thus, barriers may not be generalizable to other regions. The patient’s health literacy levels were partially obtained, 27 out of 36; hence, more granular data would be needed to analyze whether the reported challenges varied by health literacy level throughout the phases of the surgical journey. In addition, the healthcare culture in this region may not be representative of all regions in the United States. Third, the majority of physicians interviewed were surgeons. Therefore, the data may reflect more of the surgeons’ perspectives than other providers involved in cancer care.

## CONCLUSIONS

In the Deep South, low health literacy creates significant barriers for patients with GI cancer undergoing surgical treatment. Health literacy impacts each stage of the surgical journey and may contribute to delays in diagnosis, refusing recommended care, difficulty understanding the diagnosis, and delays in seeking care if a postoperative complication occurs. Improved instructions, use of visual aids, and teach-back methods were identified as 3 actionable ways to overcome these barriers. Increasing provider understanding of health literacy may further improve provider communication with patients. Lastly, the identification of barriers with administrative tasks further highlights another actionable target for interventions. Future directions aimed at improving the quality of cancer care for these patients should focus on implementing and testing interventions to help patients with low health literacy better understand and play an active role in their care.

## ACKNOWLEDGEMENTS

The research was supported by the Participant Recruitment and Assessment Shared Resource of the O’Neal Comprehensive Cancer Center (P30CA013148). The authors would like to acknowledge providers from the following institutions for participation in the study: University of Alabama at Birmingham, Infirmary Health, Mitchell Cancer Institute, North Mississippi Medical Center, Russell Medical Center, University of Mississippi Medical Center, and Whitfield Regional Hospital.

## Supplementary Material
