# Supplementary material for: The Role of Health Literacy in Gastrointestinal Cancer Patients’ Surgical Journey: A Qualitative Study
Source: Ann Surg Open. 2026 Jan 14;7(1):e643. doi: 10.1097/AS9.0000000000000643 (PMC13016180; doi:10.1097/AS9.0000000000000643)
Supplement: Supplementary file 1 [file as9-7-e643-s001.pdf]

Supplementary Table 1. Interview guide for providers (focused on the role of health literacy).

Interview Guide for Providers	
**Preoperative phase of surgical care**	
**Question 1**	**In your experience, what are some of the factors or circumstances that make access to surgery easy or difficult?**
Probe	You mentioned some circumstances related to the patient. Please tell me more about:<br>Not willing to get surgery, education and literacy, inability to understand and follow instructions, etc.
Probe	You mentioned some circumstances related to the doctors, hospitals or health system in general. Please tell me more about:<br>Patient instructions, patient/provider communication, etc.
**Perioperative phase of surgical care**	
**Question 2**	**Now let's talk about the day patients have surgery. First describe what successful surgery means from your point of view**
Probe	What is most important for you as a provider when seeing a patient through surgery?
**Question 3**	**In your opinion, what are some of the factors that may impact whether patients receive quality surgery?**
Probe	You mentioned some circumstances related to the patient. Please tell me more about:<br>Will you tell me more about the ability of patients to understand and follow instructions, and how that affects whether they get quality surgery?
**Postoperative phase of surgical care**	
**Question 4**	**Now let's talk about the day after surgery, and when patients leave the hospital. Please describe what quality discharge means:**
Probe	What is most important for you as a provider when discharging a patient?<br>What is the most important for you as a provider when following up with a patient after surgery?
**Question 5**	**What are the factors that may impact whether patients receive quality discharge and follow-up?**
Probe	How well do you think the system prepares patients for discharge?<br>Will you share examples of patients who may not have received quality discharge and follow-up and what circumstances were at play?<br>Which of the factors we discussed earlier about the period before surgery and the day of surgery, also affect the quality of discharge and follow-up?<br>Will you tell me more about the ability of patients to understand and follow instructions, and how it affects their post-surgery care?
**Question 6**	**What needs to change at the health system level, to ensure all patients first have access to surgery, and then have quality surgery and follow-up care?**
Probe	What would you consider effective and efficient system to ensure all patients have quality surgery they need?
**Question 7**	**Lastly, is there anything else you would add that would be important to consider when thinking about how to improve surgical care for GI cancer patients?**

Supplementary Table 2. Interview guide for survivors who had surgery as part of their care (focused on the role of health literacy).

**Interview Guide for Survivors (had surgery as part of their care)**	
**Preoperative phase of surgical care**	
**Question 1**	**Please tell me about how you were diagnosed with cancer, and the medical care you received after that moment.**
Probe	Would you tell me who discussed the treatment with you? What treatments were discussed and what did you receive?<br>Did you consider, or receive alternative treatments other than what was provided by the clinics or hospitals?
**Question 2**	**What thoughts went through your mind at the time after hearing the treatment recommended and that you needed surgery?**
Probe	What was your understanding of why the surgery was recommended?<br>What were your thoughts about what surgery would do for you?
**Question 3**	**Would you tell me the reasons why you went to or chose that specific hospital where you had surgery?**
Probe	What did you know at that time about hospitals where to have your type of surgery?<br>What did you know or found out about surgeons?
**Question 4**	**I would like for you to think about the medical visits you had during the time before surgery. Imagine yourself going into the buildings, the clinic, and the doctors, nurses, and staff you saw or interacted with. What experiences, feelings or interactions stood out for you in a positive or negative way?**
Probe	How would you describe the interactions with doctors, nurses, or other staff and how they made you feel?<br>How would you describe the clinic in terms of how you felt when you were there?
**Question 5**	**Please tell me how easy or difficult it was to get surgery, and why. For example, think about finding a date, scheduling, or confirming and coordinating surgery.**
Probe	What were the specific needs (if any) related to your family, work, or health, that you need to work around?<br>What were the specific needs (if any) that you needed the surgical team to work around?
**Perioperative phase of surgical care**	
**Question 6**	**Would you please describe how you prepared for surgery? Think about the instructions you were given, what you had to do, and who helped you.**
	Please tell me about which health care provider or other hospital staff helped you in preparing for surgery and how.
**Question 7**	**As you did before, please tell me about your experiences, feelings or interactions that stood out for you in a positive or negative way from your time in hospital.**
Probe	How would you describe the interactions with doctors, nurses, or other staff and how they made you feel?

	How would you describe entering the hospital and staying at the hospital in terms of how you felt physically and emotionally?
**Postoperative phase of surgical care**	
**Question 8**	**Please describe now what happened in the days after the surgery when you were still in the hospital.**
Probe	If you remember, please tell me about how many days after surgery you started eating or walking, or how pain was managed.<br>How easy or difficult were the days after surgery for you, and why?<br>How did the hospital staff and doctors make sure you were comfortable and recovering well?<br>What experiences stood out for you in a positive or negative way from this time?
**Question 9**	**Please tell me about the instructions you were given at discharge, and how easy or difficult it was to understand these instructions.**
Probe	What were you told to do once you left the hospital to take care of yourself?<br>What were you told about when to come back to see the doctor or which doctor to see?
**Question 10**	**How easy or difficult was it to follow the instructions you were given for the time after the surgery?**
Probe	How easy or difficult was it to see your doctor after surgery?<br>Will you share some examples in which your doctors or their staff followed-up with you after you left the hospital?<br>What can you tell me about the support you had at home?
**Question 11**	**Can you describe how you felt at the follow-up visits you had after you left the hospital? What experiences, feelings or interactions stood out for you in a positive or negative way?**
Probe	How would you describe the interactions with doctors, nurses and staff at this time?<br>How would you describe how you felt going into the clinic or joining the call with your doctor?
**Question 12**	**Through the overall surgery time, would you say you received the best care possible in a respectful manner?**
Probe	Please tell me more about why you felt you [received] [did not receive] the best care possible.<br>Please tell me more about what made you feel [respected] [not respected].
**Question 13**	**Apart from what we discussed, are there any other things in your experience that would make the surgery process better for patients with [participant's cancer]?**
